# A Real‐Time Prospective Evaluation of the Prognostic Accuracy of SIRS, MEWS, NEWS2 and qSOFA in Predicting ICU Admission and Mortality in an Emergency Department: Implications for Nursing Practice

**DOI:** 10.1111/nicc.70302

**Published:** 2026-01-08

**Authors:** Dimitrios Xythalis, Maria Kalafati, Meropi Mpouzika, Vassiliki Karra, Vasileios Kaldis, Konstantinos Tsekouras, Aikaterini Sofianou, Alexandros Mihopoulos, Anastasios Ioannidis, Styliani Tziaferi

**Affiliations:** ^1^ Department of Nursing University of Peloponnese Tripoli Greece; ^2^ Department of Nursing National and Kapodistrian University of Athens Athens Greece; ^3^ Department of Nursing, School of Health Sciences Cyprus University of Technology Limassol Cyprus; ^4^ Sismanoglio General Hospital of Attica Maroussi Greece; ^5^ Department of Medicine National and Kapodistrian University of Athens Athens Greece

**Keywords:** Early Warning Score, emergency service, hospital, prognosis, qSOFA, sepsis

## Abstract

**Background:**

Early identification of emergency department (ED) patients with suspected infection who are at risk for adverse outcomes is a core nursing responsibility during triage and early observation. Scoring systems vary in sensitivity and specificity, with limited prospective, real‐time comparisons in Greek EDs.

**Aim:**

To compare the prognostic accuracy of SIRS, MEWS, NEWS2 and qSOFA for predicting prolonged ICU stay (≥ 3 days) and 28‐day in‐hospital mortality and to assess performance across clinically relevant subgroups that shape nursing decision‐making.

**Study Design:**

Prospective observational study in the ED of a public general hospital in Athens, Greece. Consecutive adults (≥ 18 years) with suspected infection were enrolled. Scores were calculated in real time at triage by trained ED nurses using only presentation data. Primary outcomes were ICU stay ≥ 3 days and 28‐day in‐hospital mortality. Discrimination was assessed by AUROC (95% CI) with DeLong pairwise comparisons; prespecified subgroups included age (≥ 65 years), comorbidities, and COPD.

**Results:**

Five hundred and thirty patients who met the inclusion criteria were analysed (mean age 63.7 ± 18.5 years; 53.96% female). For predicting ICU ≥ 3 days, NEWS2 demonstrated the highest sensitivity, NPV and AUROC (0.72) with no significant differences compared to MEWS or qSOFA, but better than SIRS (*p* = 0.001). For 28‐day in‐hospital mortality, qSOFA and NEWS2 achieved the highest AUROC (0.79 and 0.77, respectively), both significantly outperforming SIRS and MEWS. NEWS2 maintained the highest sensitivity and NPV, whereas qSOFA showed the highest specificity and PPV. In subgroup analyses, comorbid patients exhibited elevated risk even with negative NEWS2/MEWS; qSOFA‐positive younger patients (< 65) had higher ICU admission; COPD was associated with higher mortality in qSOFA‐negative and MEWS‐negative strata.

**Conclusions:**

NEWS2 and qSOFA outperformed SIRS and MEWS. NEWS2 is well suited for ruling out risk (very high sensitivity/NPV), whereas qSOFA provides more specific identification of high‐risk patients.

**Relevance to Clinical Practice:**

NEWS2 can help nurses quickly rule out high‐risk infection cases, whereas qSOFA highlights patients most likely to need urgent escalation or ICU care. Using both scores together supports faster triage decisions and better allocation of monitoring and critical care resources.

## Impact Statements

- What is known about the topic
  ○ Sepsis screening tools such as SIRS, MEWS, NEWS2 and qSOFA are used to detect deterioration in patients with suspected infection, yet their prognostic accuracy varies between settings.
  ○ Most comparative studies are retrospective and lack real‐time data from emergency departments, especially within Greek healthcare systems.
  ○ No consensus exists on which score offers the best balance of sensitivity and specificity for early triage and outcome prediction.
- What this paper adds
  ○ Provides one of the first prospective, real‐time evaluations of these four tools in a Greek ED, with all scores calculated by nurses at triage.
  ○ Demonstrates that NEWS2 offers superior sensitivity and negative predictive value for ruling out severe outcomes, whereas qSOFA shows higher specificity for identifying high‐risk patients.
  ○ Highlights nursing implications, advocating combined NEWS2–qSOFA use for rapid triage, improved escalation decisions and better allocation of ICU resources.

## 1 Introduction

Early identification of patients with suspected infection who are at risk for adverse outcomes, such as death or prolonged Intensive Care Unit (ICU) stay, remains a critical challenge in emergency care [1]. Nurses are often the first healthcare professionals to assess patients, detect subtle physiological changes and initiate escalation protocols.

Given the rapid and unpredictable progression of infection‐related complications, including sepsis and the time‐sensitive nature of effective treatment, early recognition through accurate scoring systems is crucial for improving clinical outcomes. For nurses, these tools not only guide decision‐making but also structure communication with physicians, prioritise monitoring and support safe allocation of limited Emergency Department (ED) resources [2, 3, 4].

The ED, as the primary entry point to acute care, plays a critical role in the initial evaluation and triage of patients with suspected infection [5].

Several bedside scoring tools have been developed to support clinicians in detecting deterioration. The Systemic Inflammatory Response Syndrome (SIRS) criteria, despite their widespread use, have been criticised for low specificity and over‐triage [6]. Nevertheless, they may identify deterioration earlier than qSOFA, offering time‐sensitive value [7]. The Modified Early Warning Score (MEWS) [8, 9] and National Early Warning Score 2 (NEWS2) [10] are track‐and‐trigger early warning systems widely used in nursing practice, with NEWS2 mandated in the United Kingdom [11]. NEWS2 is sensitive but may overestimate risk in chronic conditions. qSOFA, introduced in Sepsis‐3, offers higher specificity but limited sensitivity, potentially missing early sepsis cases [12, 13].

These tools are designed not for diagnosis but to support early decision‐making by identifying patients with suspected infection at risk of poor outcomes such as ICU admission or in‐hospital mortality. Debate remains regarding their comparative prognostic value [14, 15]. Most studies are retrospective [16, 17, 18] and limited by documentation variability and outcome definitions [19, 20]. Consequently, international sepsis guidelines avoid endorsing a single tool, citing concerns about external validity and predictive accuracy [21].

Prospective real‐time comparisons remain scarce [19], especially in EDs where these systems are not systematically applied. In Greece, no prospective study has evaluated their comparative performance, despite NEWS2 and MEWS being widely used elsewhere in Europe and the United States [10, 22, 23].

## 2 Aim and Objectives of the Study

The aim of this study was to directly compare the performance of four widely used sepsis‐related prognostic scoring systems—SIRS, MEWS, NEWS2 and qSOFA—in adult patients presenting to the emergency department (ED) with suspected infection.

Specifically, the objectives were to (a) assess and compare the prognostic accuracy of these scoring systems in predicting two clinically relevant outcomes, prolonged intensive care unit (ICU) stay (≥ 3 days) and 28‐day in‐hospital mortality and (b) examine their predictive performance within key clinical subgroups, including age, comorbidity and chronic respiratory disease, to explore factors influencing score validity and applicability in routine ED practice.

## 3 Design and Methods

### 3.1 Study Design and Setting

This prospective observational study was conducted in the ED of a public hospital in Athens, Greece (430 beds). All scoring tools were applied and calculated at the triage stage, immediately upon patient arrival, by nurses trained in their standardised use. This approach ensured consistency and minimised measurement bias. Reporting followed STROBE guidelines [24].

### 3.2 Sample of the Study

Inclusion criteria: adults (≥ 18 years) presenting with clinically suspected infection and complete follow‐up data during ED stay or hospital admission. Exclusion criteria: need for immediate CPR or pregnancy. Adult focus was justified as the scores were validated in this population [9, 10, 12, 25].

Guidance suggests ≥ 100–200 cases are sufficient for AUROC precision [26, 27]. Our cohort exceeded this and was larger than several previous prospective comparisons [19, 20].

### 3.3 Data Collection Process

Data collection was conducted over a one‐year period, from December 1, 2023, to December 1, 2024. Upon arrival of each patient to the ED triage, data were collected prospectively by the trained nurses.

A structured form, pilot‐tested for clarity and feasibility, recorded: (a) demographics (age, sex), (b) relevant history, (c) arrival and triage times, (d) vital signs and mental status, (e) suspected infection site and oxygen method, (f) laboratory results, and (g) scoring results.

Scoring systems definitions:

- SIRS (Systemic Inflammatory Response Syndrome): Score range 0–20. Assesses the following parameters: (a) body temperature > 38°C or < 36°C, (b) heart rate > 90 bpm, (c) respiratory rate > 20 breaths/min or PaCO_2_ < 32 mmHg, and (d) white blood cell (WBC) count > 12 000/mm^3^, < 4000/mm^3^, or > 10% immature (band) forms. A score ≥ 2 indicates a positive SIRS result suggestive of systemic inflammation [25].
- MEWS (Modified Early Warning Score): Score range 0–17. Evaluates six physiological variables: temperature, heart rate, systolic blood pressure (SBP), respiratory rate, level of consciousness using AVPU (Alert, Verbal, Pain, Unresponsive) scale and urine output. Urine output was excluded in this ED‐based study, consistent with common practice [8, 9]. A score ≥ 4 indicates increased clinical risk.
- NEWS2 (National Early Warning Score 2): Score range 0–20. Considers seven variables: respiratory rate, oxygen saturation (SpO_2_), need for supplemental oxygen, SBP, heart rate, temperature and neurological status (AVPU scale). NEWS2 includes a specific SpO_2_ scoring modification for patients with chronic hypercapnic respiratory failure, targeting a range of 88%–92% [10]. A score ≥ 5 triggers an urgent clinical alert.
- qSOFA (quick Sequential Organ Failure Assessment): Score range 0–3. Includes three criteria: altered mental status (Glasgow Coma Scale < 15), SBP ≤ 100 mmHg and respiratory rate ≥ 22 breaths/min [12]. A score ≥ 2 suggests increased risk of poor outcomes, including ICU admission and mortality.

Score positivity was defined as meeting or exceeding the established threshold for each scoring tool (SIRS ≥ 2, MEWS ≥ 4, NEWS2 ≥ 5, qSOFA ≥ 2).

Due to the study's prospective design and real‐time data collection, missing data were minimal. Missing WBC counts (17.3% of patients) were scored as normal for SIRS. Follow‐up included hospital admission, ICU outcomes and 28‐day mortality.

### 3.4 Study Procedures and Outcome Measures

Patients were enrolled using a consecutive sampling method. All eligible patients presenting during the study period were screened for inclusion, based on clinical suspicion of infection, as determined by the triage physician. This reflects real‐world ED practice, where no scoring system is formally used to initiate suspicion of infection [28, 29, 30]. Instead, inclusion was guided by a combination of subjective symptoms, objective clinical findings and relevant medical history. The triage physician making this determination varied by shift and could belong to any medical specialty, based on the ED's rotating duty schedule. To reduce variability, based on arrival and triage times, only patients evaluated within 1 h of ED arrival were included. Patients assessed beyond this time frame were excluded to minimise time‐dependent distortion of scoring system values [31, 32].

In cases where the presence of infection was initially uncertain, patients were provisionally included and later excluded if alternative diagnoses were confirmed, either during their ED stay or post‐admission. When infection status remained uncertain, the case was discussed with the attending physician, whose clinical judgement guided final inclusion or exclusion.

For patients discharged from the ED without additional diagnostic testing, inclusion was based on clinical findings and the physician's judgement alone, with discharge as the final outcome since no further monitoring of their health status was possible.

Infection confirmation was based on one or more of the following criteria (in order of priority):

- Diagnosis coded as infection using International Classification of Diseases, 10th Revision (ICD‐10) [33]
- Positive microbiological cultures within 24 h of presentation
- Pathogen identification via molecular or rapid antigen detection methods (e.g., influenza test)
- Urine microscopy shows significant pyuria
- Abnormal WBC counts (> 12 000/mm^3^, < 4000/mm^3^ or > 10% immature neutrophils)
- Radiographic evidence consistent with infection
- Initiation of antibiotics within the first 24 h

The criteria for infection were established based on the rationale of the study, the commonly accepted standards in everyday clinical practice, and those used by the European Centre for Disease Prevention and Control (ECDC) [34]. WBC count was selected due to its established use as a criterion for infection in the SIRS criteria.

ICU stay of ≥ 3 days and 28‐day in‐hospital mortality were selected as the study's primary outcomes, based on their clinical relevance and alignment with previously validated prognostic models in patients with suspected infection [35, 36, 37]. Follow‐up relied on hospital records.

### 3.5 Data Analysis

Analyses included (1) individual score performance, (2) pairwise AUROC comparisons using the DeLong test and (3) subgroup analyses (age ≥ 65, comorbidities, COPD). Prognostic metrics: sensitivity, specificity, PPV, NPV, AUROC with 95% CI. Differences in subgroup outcomes were assessed using chi‐squared or Fisher's exact tests. A two‐tailed *p* value of < 0.05 was considered statistically significant.

All statistical analyses were conducted using IBM SPSS Statistics (version 26.0) and MedCalc (version 20.1) software.

## 4 Ethical Considerations

The study was approved by the Hospital's Board of Directors (November 2, 2023, 22264/09‐11‐2023). No additional interventions beyond routine ED care were performed, and written informed consent was not required, in accordance with national regulations and consistent with similar observational studies [17, 35]. All patient data were handled in compliance with applicable data protection laws and institutional policies.

## 5 Results

### 5.1 Baseline Patient Characteristics, Scoring Systems and Clinical Outcomes

A total of 918 patients presenting to the ED were screened for suspected infection. Of these, 566 met initial inclusion criteria, and 530 were ultimately included in the final analysis after excluding 36 patients whose final diagnoses did not confirm infection.

The mean age of the included cohort was 63.7 years (SD ±18.5; range 18–101 years), and 53.96% were female. The most frequent site of infection was the lower respiratory tract (55.6%), followed by the urinary tract (24.9%). A summary of baseline demographic and clinical characteristics is presented in Table 1.

**TABLE 1:** Baseline patient characteristics, scoring systems and clinical outcomes.

Variable	Value
Age (years)	Mean: 63.7 (SD ±18.5)
Sex	Male: 48.7% (*n* = 258) Female: 51.3% (*n* = 272)
Site of infection	Lower respiratory tract: 55.7% (*n* = 295) Urinary system: 17.5% (*n* = 93) Unknown: 10.6 (*n* = 56) Digestive system: 6.4% (*n* = 34) Multiple systems: 2.5% (*n* = 13) Soft tissue: 2.1% (*n* = 11) Oral cavity: 1.5% (*n* = 8) Upper respiratory Tract: 1.3% (*n* = 7) Bloodstream: 1.3% (*n* = 7) Εyes nose throat: 0.7% (*n* = 4) Eyes: 0.2% (*n* = 1) Cardiopulmonary system: 0.2% (*n* = 1)
Scoring systems positivity	SIRS ≥ 2: 52.8% (*n* = 280) MEWS ≥ 4: 25.3% (*n* = 134) NEWS2 ≥ 5: 48.3% (*n* = 256) qSOFA ≥ 2: 18.3% (*n* = 97)
Hospital admission	Yes: 49.6% (*n* = 263) No: 51.4% (*n* = 267)
ICU stay of ≥ 3 days	Yes: 4.3% (*n* = 23) No: 95.7% (*n* = 507)
Clinical status at Day 28 among hospitalised patients	Discharged: 73.4% (*n* = 193) Still hospitalised: 9.1% (*n* = 24) Deceased: 17.5% (*n* = 46)
28‐day in‐hospital mortality (total patients)	Yes: 8.7% (*n* = 46) No: 91.3% (*n* = 484)

### 5.2 Scoring Systems Positivity Rates

The proportion of patients classified as positive for each prognostic scoring system was determined according to established threshold values: SIRS ≥ 2, MEWS ≥ 4, NEWS2 ≥ 5 and qSOFA ≥ 2. Positivity was defined as a score equal to or exceeding these cut‐off points, indicating a level of physiological derangement associated with increased risk of adverse outcomes. Score positivity rates were as follows: SIRS ≥ 2 in 52.8%, MEWS ≥ 4 in 25.3%, NEWS2 ≥ 5 in 48.3% and qSOFA ≥ 2 in 18.3% of patients.

### 5.3 Predictive Accuracy of Scoring Systems for ICU Stay ≥ 3 Days

The performance metrics for predicting ICU stays of ≥ 3 days are presented in Table 2. NEWS2 demonstrated the highest discriminative ability (AUROC = 0.72), followed by MEWS and qSOFA, while SIRS had poor performance. NEWS2 also provided the highest sensitivity and NPV, indicating its usefulness for ruling out patients unlikely to require prolonged ICU care, whereas qSOFA and MEWS achieved higher specificity, supporting their role in identifying higher‐risk cases.

**TABLE 2:** Predictive accuracy of scoring systems for ICU stay ≥ 3 days.

Scoring system	Sensitivity (95% CI)	Specificity (95% CI)	PPV (95% CI)	NPV (95% CI)	AUROC (95% CI)
SIRS	86.96% (67.9–95.5)	41.81% (37.6–46.2)	6.35% (4.1–9.6)	98.60% (96.0–99.5)	0.6439 (0.6031–0.6847)
MEWS	65.22% (44.9–81.2)	76.50% (72.6–80.0)	11.19% (6.9–17.6)	97.97% (96.1–99.0)	0.7088 (0.6702–0.7474)
NEWS2	91.30% (73.2–97.6)	53.65% (49.3–57.9)	8.20% (5.4–12.2)	99.26% (97.4–99.8)	0.7248 (0.6868–0.7628)
qSOFA	56.52% (36.8–74.4)	82.80% (79.3–85.9)	13.00% (7.8–21.0)	97.67% (95.8–98.7)	0.6968 (0.6578–0.7358)

**FIGURE 1:**
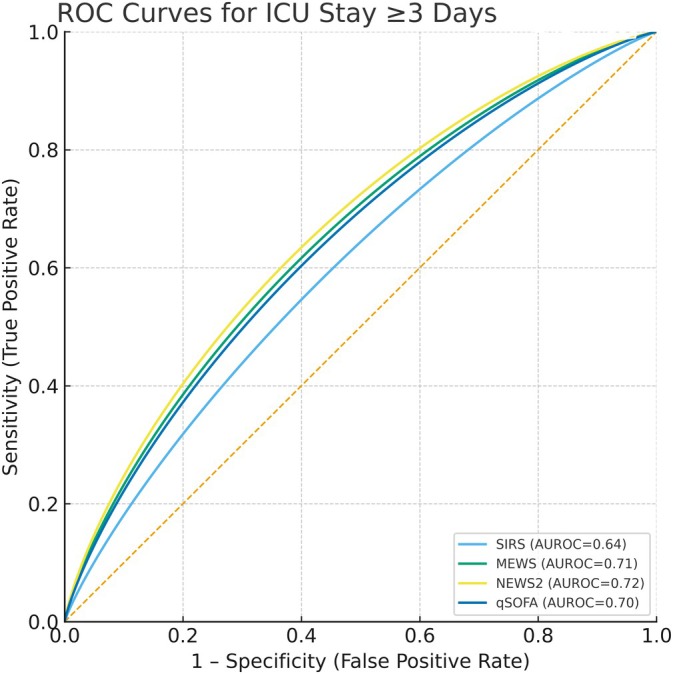
ROC curves for ICU stay ≥ 3 days.

Pairwise AUROC comparisons (Table 3, Figure 1) confirmed NEWS2's superiority over SIRS (*p* = 0.001), with no significant differences between NEWS2, MEWS and qSOFA.

**TABLE 3:** AUROC pairwise comparison summary for ICU stay ≥ 3 days.

Comparison	*p*
NEWS versus SIRS	0.001
NEWS2 versus MEWS	0.563
NEWS2 versus qSOFA	0.314
MEWS versus SIRS	0.024
MEWS versus qSOFA	0.688
qSOFA versus SIRS	0.066

### 5.4 Predictive Accuracy of Scoring Systems for 28‐Day In‐Hospital Mortality

The performance metrics of each scoring system for predicting 28‐day in‐hospital mortality are presented in Table 4.

**TABLE 4:** Predictive accuracy of scoring systems for 28‐day in‐hospital mortality.

Score	Sensitivity (95% CI)	Specificity (95% CI)	PPV (95% CI)	NPV (95% CI)	AUROC (95% CI)
SIRS	89.13% (77.4–95.4)	43.39% (38.7–48.2)	13.02% (9.7–17.1)	97.67% (94.6–99.0)	0.6626 (0.6224–0.7028)
MEWS	60.87% (44.7–75.0)	78.10% (73.8–82.0)	20.90% (14.7–28.7)	95.45% (92.4–97.4)	0.6949 (0.6557–0.7341)
NEWS2	97.83% (87.9–99.9)	56.40% (51.5–61.2)	17.58% (13.4–22.7)	99.64% (97.8–100)	0.7712 (0.7355–0.8069)
qSOFA	71.74% (55.4–84.3)	86.16% (82.4–89.3)	33.00% (24.1–43.2)	96.98% (94.6–98.4)	0.7895 (0.7548–0.8242)

qSOFA achieved the highest overall discriminative performance (AUROC = 0.79), followed closely by NEWS2 (AUROC = 0.77). NEWS2 maintained the highest sensitivity and NPV, whereas qSOFA had the highest specificity and PPV.

**FIGURE 2:**
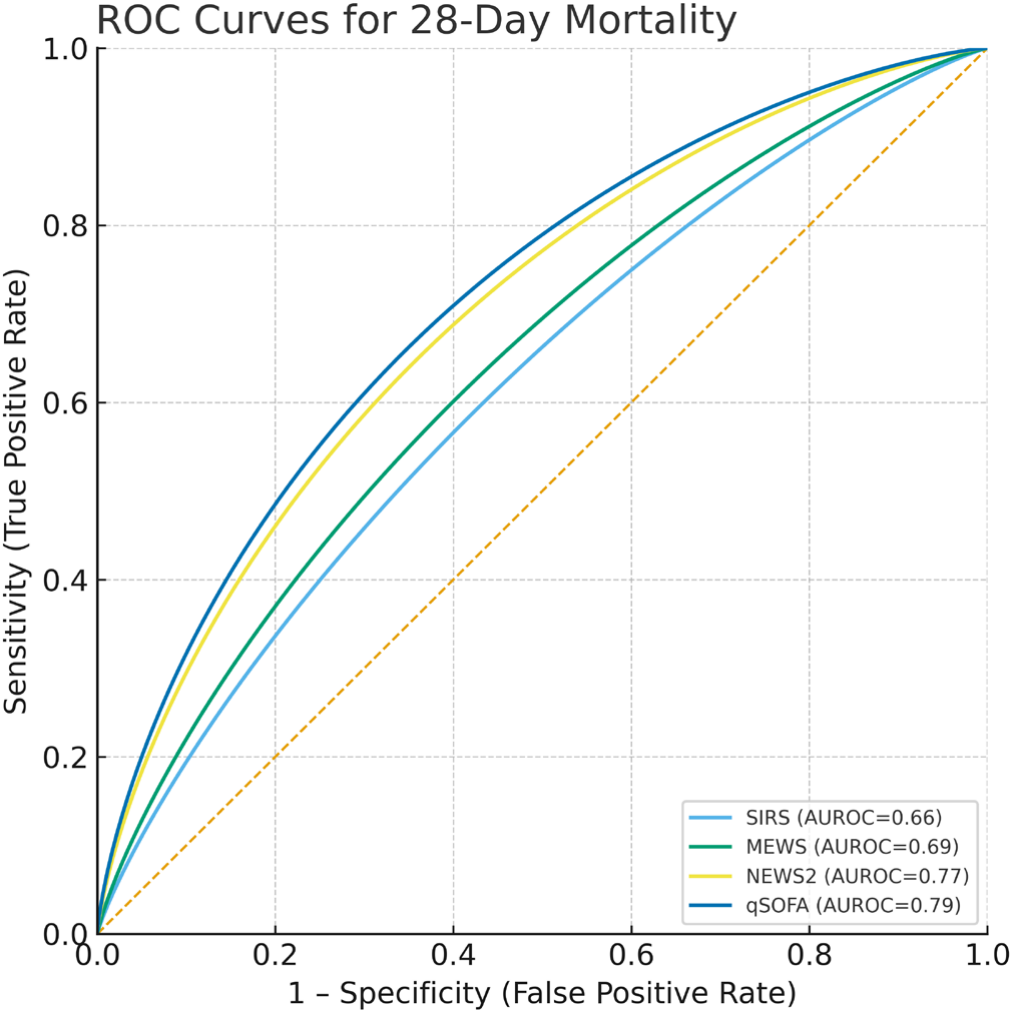
ROC curves for 28‐day in‐hospital mortality.

DeLong tests (Table 5, Figure 2) showed both NEWS2 and qSOFA significantly outperformed SIRS and MEWS, with no significant difference between NEWS2 and qSOFA (*p* = 0.15).

**TABLE 5:** AUROC pairwise comparison summary for 28‐day in‐hospital mortality.

Comparison	*p* value
NEWS2 versus SIRS	0.001
qSOFA versus SIRS	0.0002
NEWS2 versus MEWS	0.003
qSOFA versus MEWS	0.003
qSOFA versus NEWS2	0.15

### 5.5 Subgroup Analysis

Subgroup analyses explored whether the prognostic performance of the scoring systems differed by age, comorbidity and COPD status.

#### 5.5.1 Prediction of ICU Stay ≥ 3 Days

Detailed statistics are presented in Table 6. Comorbidities, age and COPD influenced score performance to varying degrees. Among patients with comorbidities, higher ICU admission rates were observed even when standard thresholds of NEWS2 or MEWS were not met, suggesting that these scores may underestimate ICU risk in multi‐morbid patients. SIRS positivity was also more frequently associated with ICU admission in those with comorbidities, whereas the qSOFA pattern was less consistent.

**TABLE 6:** Subgroup analysis for ICU stay of ≥ 3 days.

Scoring system	Score status	Subgroup	ICU ≥ 3 in + subgroup = yes (%)	ICU ≥ 3 in + subgroup = no (%)	*p*
qSOFA	Positive	Comorbidity	14.94	0.0	0.3655
SIRS	Positive	Comorbidity	8.23	1.2	0.0472*
NEWS2	Positive	Comorbidity	9.3	2.63	0.2914
MEWS	Positive	Comorbidity	14.0	3.12	0.1716
qSOFA	Negative	Comorbidity	3.85	0.51	0.0494*
SIRS	Negative	Comorbidity	3.33	0.0	0.1469
NEWS2	Negative	Comorbidity	4.33	0.52	0.0309*
MEWS	Negative	Comorbidity	3.64	0.0	0.0286*
qSOFA	Positive	COPD	21.05	11.11	0.435
SIRS	Positive	COPD	6.33	6.36	1.0
NEWS2	Positive	COPD	8.05	8.28	1.0
MEWS	Positive	COPD	16.67	9.62	0.453
qSOFA	Negative	COPD	3.8	1.99	0.5839
SIRS	Negative	COPD	10.53	0.51	0.0114*
NEWS2	Negative	COPD	0.0	0.76	1.0
MEWS	Negative	COPD	2.94	1.83	0.9048
qSOFA	Positive	Age ≥ 65	9.89	50.0	0.0075*
SIRS	Positive	Age ≥ 65	5.61	8.08	0.5595
NEWS2	Positive	Age ≥ 65	6.6	16.28	0.0718
MEWS	Positive	Age ≥ 65	10.31	13.51	0.8262
qSOFA	Negative	Age ≥ 65	2.83	1.84	0.7209
SIRS	Negative	Age ≥ 65	3.41	0.0	0.1345
NEWS2	Negative	Age ≥ 65	1.1	0.55	1.0
MEWS	Negative	Age ≥ 65	2.42	1.59	0.82

* *p* < 0.05.

A paradoxical pattern was observed in qSOFA‐positive patients: those under 65 years had significantly higher ICU admission rates than those aged ≥ 65 years. This may reflect more aggressive ICU admissions in younger high‐risk patients. No other age‐stratified comparisons reached statistical significance.

For COPD, an increased ICU stay risk appeared mainly among SIRS‐negative individuals, indicating that chronic respiratory disease may mask physiological derangements otherwise captured by SIRS criteria.

#### 5.5.2 Prediction of 28‐Day In‐Hospital Mortality

Detailed statistics are presented in Table 7. Mortality analyses revealed that comorbidity and advanced age significantly amplified risk across nearly all scoring systems. Patients with comorbidities had markedly higher mortality both when scores were positive and, importantly, when they were negative—again underscoring potential false‐negative limitations of NEWS2, MEWS and qSOFA in complex chronic illness.

**TABLE 7:** Subgroup analysis for 28‐day in‐hospital mortality.

Scoring system	Score status	Subgroup	Mortality in subgroup = yes (%)	Mortality in subgroup = no (%)	*p*
qSOFA	Positive	Comorbidity	36.36	9.09	0.14165
SIRS	Positive	Comorbidity	17.32	1.2	0.0004*
NEWS2	Positive	Comorbidity	20.37	2.56	0.014*
MEWS	Positive	Comorbidity	26.73	3.12	0.0092*
qSOFA	Negative	Comorbidity	5.56	0.0	0.0022*
SIRS	Negative	Comorbidity	5.49	0.0	0.029*
NEWS2	Negative	Comorbidity	0.94	0.0	0.816
MEWS	Negative	Comorbidity	8.14	0.0	0.0003*
qSOFA	Positive	Age ≥ 65	34.78	12.5	0.3715
SIRS	Positive	Age ≥ 65	18.6	1.0	0.0
NEWS2	Positive	Age ≥ 65	20.66	2.33	0.0078*
MEWS	Positive	Age ≥ 65	27.84	2.7	0.0031*
qSOFA	Negative	Age ≥ 65	6.13	0.0	0.0006*
SIRS	Negative	Age ≥ 65	5.62	0.0	0.0256*
NEWS2	Negative	Age ≥ 65	1.1	0.0	0.721
MEWS	Negative	Age ≥ 65	8.7	0.0	0.0001*
qSOFA	Positive	COPD	52.63	28.4	0.0799*
SIRS	Positive	COPD	20.25	10.59	0.0438*
NEWS2	Positive	COPD	20.69	15.98	0.4442
MEWS	Positive	COPD	30.0	18.27	0.2554
qSOFA	Negative	COPD	10.13	1.42	0.0002*
SIRS	Negative	COPD	10.53	1.53	0.0916
NEWS2	Negative	COPD	0.0	0.38	1.0
MEWS	Negative	COPD	13.24	2.74	0.0005*

* *p* < 0.05.

Older adults (≥ 65 years) demonstrated substantially higher mortality across all scoring systems, regardless of score status, confirming age as a dominant prognostic determinant.

In COPD, excess mortality persisted even when qSOFA or MEWS were negative, emphasising that chronic respiratory disease can attenuate score sensitivity and warrant cautious interpretation.

## 6 Discussion

In this prospective, real‐time evaluation of adult patients with suspected infection presenting to a Greek ED, we compared the prognostic accuracy of four widely used sepsis‐related scoring systems (SIRS, MEWS, NEWS2 and qSOFA) for two clinically relevant outcomes: prolonged ICU stay (≥ 3 days) and 28‐day and in‐hospital mortality. Our findings highlight important differences in the discriminative abilities of these scores and provide practical insights into their strengths and limitations across key patient subgroups.

### 6.1 Overall Prognostic Performance

Our findings provide important implications for nursing practice. Across the full cohort, both NEWS2 and qSOFA demonstrated the most balanced prognostic accuracy, supporting international evidence that no single scoring system achieves both high sensitivity and high specificity across all settings [20, 38, 39].

NEWS2's composite structure, which incorporates oxygen saturation, supplemental oxygen use and graded vital‐sign weighting, enables it to detect early physiological instability even before overt organ failure occurs. This likely explains its high sensitivity and negative predictive value observed in our cohort. In contrast, qSOFA, based solely on respiratory rate, systolic pressure, and mental status, identifies patients with already manifest organ dysfunction; consequently, it is more specific but less sensitive, a pattern consistently described in meta‐analyses by Ruan et al. [19] and Rudd et al. [40].

The lower specificity of SIRS and the intermediate performance of MEWS reflect their design origins. SIRS was intended for early inflammatory detection rather than mortality prediction, while MEWS was built for general deterioration and lacks infection‐specific triggers. Similar limitations have been documented by Sabir et al. [20] and Adegbite et al. [41], who noted that both scores tend to overclassify risk in emergency cohorts.

### 6.2 Prediction of ICU Stay ≥ 3 Days

NEWS2 outperformed other scoring systems in identifying patients who later required prolonged ICU care. This can be explained physiologically: unlike qSOFA or MEWS, NEWS2 integrates oxygenation indices and subtle changes in respiration and consciousness that often precede hemodynamic collapse. These parameters capture ‘early instability’, a hallmark of patients who will deteriorate even if organ failure is not yet established. Uffen et al. [42] emphasised that such early triage sensitivity is essential in ED pathways to trigger timely escalation and protocolised management.

However, NEWS2's advantage in sensitivity comes with lower specificity, potentially causing alert fatigue or over‐triage—an issue widely reported in ED sepsis pathways [42]. By contrast, qSOFA and MEWS, which require more pronounced physiological derangement, yield fewer false alarms but may miss evolving cases. Inter‐rater variation could also have influenced performance: small inconsistencies in mental‐status assessment or respiratory‐rate counting—tasks often performed by different nurses—can shift scores by one or two points and affect classification [40]. These findings underline the importance of continuous nursing training.

### 6.3 Prediction of 28‐Day In‐Hospital Mortality

For mortality, qSOFA achieved slightly higher discriminative ability than NEWS2, consistent with global data indicating that qSOFA is a strong predictor of death once organ dysfunction is evident [19, 40]. Its emphasis on hypotension and altered mentation captures circulatory and neurologic compromise, both late but highly prognostic markers. In contrast, NEWS2, though highly sensitive, may overestimate mortality risk in transiently hypoxic or febrile patients whose condition stabilises after initial therapy. This trade‐off mirrors findings from large reviews by Kumar et al. [39] and Zhang et al. [38], who concluded that NEWS‐type systems perform best as continuous monitoring tools, while qSOFA is preferable for identifying patients already in need of urgent intervention.

The coexistence of these complementary characteristics suggests a potential sequential approach, using NEWS2 for broad triage screening and qSOFA for targeted escalation or ICU referral, as proposed by Adegbite et al. [41].

### 6.4 Subgroup Insights

Subgroup analysis revealed that score performance is modified by patient characteristics, an observation aligned with recent calls for context‐specific score calibration.

- Comorbidity: In multi‐morbid patients, adverse outcomes occurred even with negative scores, suggesting that standard thresholds may underestimate risk when chronic disease blunts physiological responses. Future adaptations could integrate comorbidity weightings or biomarker inputs (e.g., CRP or lactate), as supported by Kumar et al. [39] who advocate multi‐parameter hybrid tools.
- Age: Older adults consistently exhibited higher mortality despite similar score distributions, reflecting reduced physiological reserve and atypical infection presentations. Zhang et al. [38] demonstrated that NEWS sensitivity declines sharply in older cohorts, a limitation also observed in our data.
- COPD: Chronic respiratory disease confounded oxygen‐based criteria and reduced the discriminatory value of NEWS2 and MEWS—issues previously noted in respiratory‐specific analyses [43, 44]. Adjusted thresholds or parallel evaluation with baseline SpO_2_ could mitigate this bias.

These patterns underscore that sepsis screening cannot rely solely on fixed cut‐offs but must be interpreted within clinical and contextual judgement, an area where nurses' ongoing patient observation adds critical value.

## 7 Implications for Nursing Practice and Research

For emergency nursing, these results highlight the need for dual‐purpose sepsis screening: NEWS2 as a sensitive triage trigger integrated into vital‐sign monitoring, and qSOFA as a confirmatory, high‐specificity escalation tool. Continuous professional education should address inter‐rater reliability, particularly in assessing respiratory rate and mental status.

Future research should examine combined or ‘augmented’ scores, such as NEWS2 plus biomarkers (CRP, lactate or D dimers) and validate them prospectively in diverse ED populations. Exploring digital or AI‐assisted triage algorithms, which dynamically weight comorbidity and age, could further optimise accuracy and reduce human error.

## 8 Strengths and Limitations

This study has several strengths, including its prospective real‐time design, relatively large sample size and inclusion of clinically relevant outcomes beyond ICU admission (≥ 3‐day ICU stay specifically and 28‐day mortality). To our knowledge, it is one of the few prospective evaluations of these scores conducted in a Greek ED. The analysis into whether there is a statistically significant difference between the AUROC values of the scoring systems in terms of their prognostic ability is something that has not been conducted in any previous research to date.

However, limitations should be acknowledged. First, the study was conducted at a single centre, which may limit generalisability to other Greek or international settings. Second, while outcomes were clinically meaningful, we did not capture intermediate endpoints such as organ support requirements.

A key limitation relates to the primary outcome of 28‐day in‐hospital mortality, as this may underestimate the true mortality burden if deaths occurred after discharge. While all outcomes were verified through hospital records and no patients were lost to follow‐up, the lack of post‐discharge mortality data introduces a potential selection bias that could influence the performance estimates of the prognostic scores. Nevertheless, considering that patients are typically not discharged unless clinically stable, it is unlikely that a significant number of deaths occurred following discharge.

Future studies should in‐corporate mechanisms to track post‐discharge mortality to more comprehensively assess outcomes. Finally, as with all prognostic scoring systems, thresholds were predefined, and alternative cutoffs might alter sensitivity/specificity trade‐offs.

## 9 AI Use Declaration

Generative Artificial Intelligence (AI) was used in a limited and fully supervised manner for figure rendering and language refinement. Specifically, the authors used ChatGPT to (i) assist with graphical rendering of ROC/AUROC figures based on our already‐computed statistics and (ii) perform grammar and clarity edits on author‐written text. No AI system generated scientific content, data, analyses or references, and AI outputs were line‐by‐line verified by the authors for accuracy and discipline‐appropriate terminology.

## 10 Conclusions

In summary, while NEWS2 and qSOFA each demonstrate valuable but complementary strengths, their performance depends on the clinical goal and patient context. NEWS2's high sensitivity supports early nurse‐led detection and safe exclusion of low‐risk cases, whereas qSOFA's specificity helps identify those at imminent risk who require escalation. Embedding both tools within a structured, education‐supported sepsis pathway aligns with global evidence and offers the most balanced approach for ED practice.

## Funding

The authors have nothing to report.

## Ethics Statement

The study was conducted in accordance with the Declaration of Helsinki and approved by the Institutional Review Board of Sismanoglio General Hospital of Attica (22 264/09‐11‐2023). All patient data were handled in compliance with applicable data protection laws and institutional policies.

## Consent

Patient consent was waived as all data were obtained as part of standard care and involved no identifiable personal information.

## Conflicts of Interest

The authors declare no conflicts of interest.

## Data Availability

The data that support the findings of this study are available from the corresponding author upon reasonable request.
